# Development of a prediction model for the acquisition of extended spectrum beta-lactam-resistant organisms in U.S. international travellers

**DOI:** 10.1093/jtm/taad028

**Published:** 2023-03-02

**Authors:** David Garrett Brown, Colin J Worby, Melissa A Pender, Ben J Brintz, Edward T Ryan, Sushmita Sridhar, Elizabeth Oliver, Jason B Harris, Sarah E Turbett, Sowmya R Rao, Ashlee M Earl, Regina C LaRocque, Daniel T Leung

**Affiliations:** Division of Infectious Diseases, University of Utah School of Medicine, Salt Lake City, UT, USA; Infectious Disease and Microbiome Program, Broad Institute of MIT and Harvard, Cambridge, MA, USA; Division of Infectious Diseases, University of Utah School of Medicine, Salt Lake City, UT, USA; Division of Epidemiology, University of Utah School of Medicine, Salt Lake City, UT, USA; Harvard Medical School, Boston, MA, USA; Travelers’ Advice and Immunization Center, Massachusetts General Hospital, Boston, MA, USA; Division of Infectious Diseases, Massachusetts General Hospital, Boston, MA, USA; Department of Medicine, Massachusetts General Hospital, Boston, MA, USA; Harvard Medical School, Boston, MA, USA; Division of Infectious Diseases, Massachusetts General Hospital, Boston, MA, USA; Department of Medicine, Massachusetts General Hospital, Boston, MA, USA; Division of Infectious Diseases, Massachusetts General Hospital, Boston, MA, USA; Division of Infectious Diseases, Massachusetts General Hospital, Boston, MA, USA; Department of Pediatrics, Harvard Medical School, Boston, MA, USA; Harvard Medical School, Boston, MA, USA; Division of Infectious Diseases, Massachusetts General Hospital, Boston, MA, USA; Department of Pathology, Massachusetts General Hospital, Boston, MA, USA; Department of Global Health, Boston University School of Public Health, Boston, MA, USA; Infectious Disease and Microbiome Program, Broad Institute of MIT and Harvard, Cambridge, MA, USA; Harvard Medical School, Boston, MA, USA; Travelers’ Advice and Immunization Center, Massachusetts General Hospital, Boston, MA, USA; Division of Infectious Diseases, Massachusetts General Hospital, Boston, MA, USA; Department of Medicine, Massachusetts General Hospital, Boston, MA, USA; Division of Infectious Diseases, University of Utah School of Medicine, Salt Lake City, UT, USA; Division of Microbiology & Immunology, University of Utah School of Medicine, Salt Lake City, UT, USA

**Keywords:** ESBL, international travel, clinical prediction, antibiotic resistance, machine learning

## Abstract

**Background:**

Extended spectrum beta-lactamase producing *Enterobacterales* (ESBL-PE) present a risk to public health by limiting the efficacy of multiple classes of beta-lactam antibiotics against infection. International travellers may acquire these organisms and identifying individuals at high risk of acquisition could help inform clinical treatment or prevention strategies.

**Methods:**

We used data collected from a cohort of 528 international travellers enrolled in a multicentre US-based study to derive a clinical prediction rule (CPR) to identify travellers who developed ESBL-PE colonization, defined as those with new ESBL positivity in stool upon return to the United States. To select candidate features, we used data collected from pre-travel and post-travel questionnaires, alongside destination-specific data from external sources. We utilized LASSO regression for feature selection, followed by random forest or logistic regression modelling, to derive a CPR for ESBL acquisition.

**Results:**

A CPR using machine learning and logistic regression on 10 features has an internally cross-validated area under the receiver operating characteristic curve (cvAUC) of 0.70 (95% confidence interval 0.69–0.71). We also demonstrate that a four-feature model performs similarly to the 10-feature model, with a cvAUC of 0.68 (95% confidence interval 0.67–0.69). This model uses traveller’s diarrhoea, and antibiotics as treatment, destination country waste management rankings and destination regional probabilities as predictors.

**Conclusions:**

We demonstrate that by integrating traveller characteristics with destination-specific data, we could derive a CPR to identify those at highest risk of acquiring ESBL-PE during international travel.

## Introduction

Alongside the global increase in the consumption of antibiotics is a subsequent rise in antimicrobial-resistant organisms (AROs).^1^ AROs are a public health threat, and recent work suggests that over 1.4 million deaths were directly attributable to infection from these agents in 2019.^2^ ARO prevalence varies by geographic region, and some international travellers are at risk of acquisition, carriage and dissemination of these organisms. Previous studies in international travellers have found that, in general, 20–40% of European/North American residents return from travel with new ARO colonization, with wider ranges observed when stratified by travel region.^3–9^ Tools to identify travellers at highest risk of ARO acquisition can help inform treatment decisions and precautionary measures.

Extended spectrum beta-lactamase (ESBL) producing *Enterobacterales* (ESBL-PE) are a concerning order of Gram-negative bacilli that are among the AROs commonly acquired by international travellers. *Enterobacterales* includes organisms such as *Escherichia coli* and *Klebsiella* species which usually asymptomatically colonize the gastrointestinal tract but may also cause infection. In many healthcare settings, empiric treatment of severe Gram-negative infections includes the use of beta-lactam antibiotics which may not appropriately treat ESBL-PE infections.^10^^,^^11^ Among patients who receive delayed or inappropriate therapy for severe Gram-negative infections, mortality rates can exceed 38%.^10^^,^^12–14^ Thus, early effective antibiotic selection is critical among patients with medium to high mortality risk.^15^

Prior studies have identified multiple risk factors for ESBL-PE acquisition in travellers. Rates vary between travel destinations, with the highest frequencies of acquisition associated with travel to Southern and Southeastern Asia.^3^^,^^4^^,^^16–18^ Gastrointestinal risk factors for travel-associated acquisition of ESBL-PE include occurrence of traveller’s diarrhoea, particularly when treated with antibiotics,^3^^,^^9^^,^^19^ as well as inflammatory bowel disease.^17^ More broadly, studies have associated traveller characteristics such as age and diet/food-related features with risk.^3^ Features detailing reasons for travelling, such as travelling for leisure or to visit friends and relatives, are also associated with acquisition risk.^19^

Clinical prediction rules (CPRs) are an established tool to aid clinicians in diagnosis and treatment and could be useful in promptly identifying individuals at risk for travel-acquired AROs. In the development of these tools, we use analytical methods that identify variables that can predict outcome, but may or may not be causally associated with risk.^20^^,^^21^ Prediction of ESBL-PE carriage in returned travellers would alert clinicians to select more appropriate initial antimicrobial therapy in the event of Gram-negative infection and may inform infection control precautions to reduce nosocomial spread. Among travellers presenting for pre-travel evaluation, a CPR may identify at-risk individuals who could benefit from further counselling on topics such as food and water safety and potential harms of inappropriate antibiotic use. In this study, we aim to derive and validate a CPR for ESBL-PE acquisition in a cohort of U.S. international travellers. We caution that the goal of this work, and the methods used, is not for causal inference, rather the development of a tool to assess risk.

## Methods

### Description of international traveller cohort and clinical data

**Figure 1:**
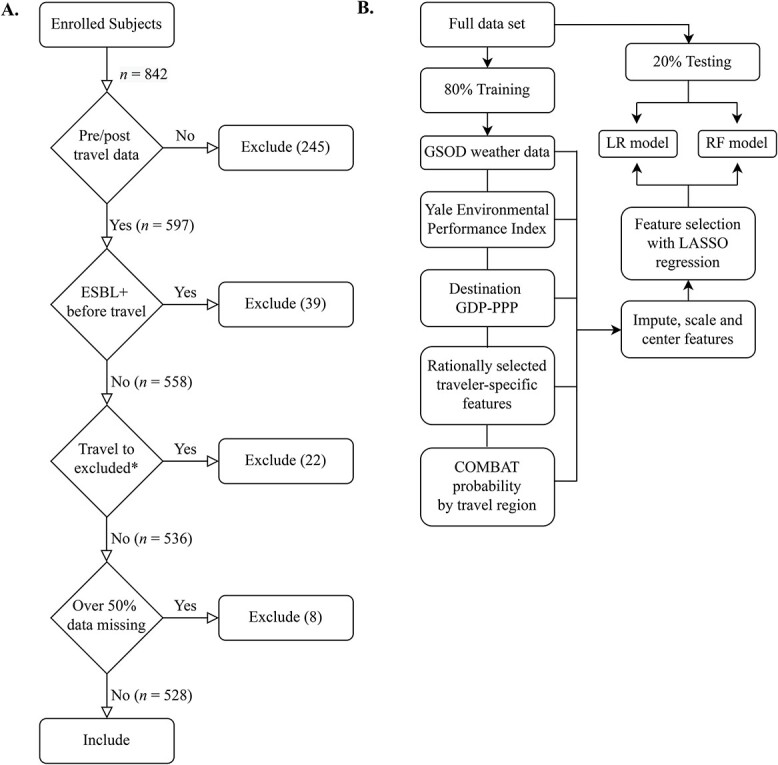
Processing of subjects and features. (A) Subjects were excluded if they did not have ESBL data before or after travel, if they were ESBL+ before travel, if significant data were missing or if they travelled to an excluded territory or country. From 842 initial subjects, 528 were included in the study. (B) Data were split into training and testing sets. Weather, selected Yale EPI rankings, regional probability derived from the COMBAT study, and traveller-specific data were imputed, scaled, centred and selected with LASSO regression. We trained LR or RF models on the training set, and then cross-validated each model on the testing set. ^*^Yale EPI and/or GDP-PPP were not available for specific destinations (detailed in methods). Travellers to these destinations were excluded

As detailed previously, we enrolled prospective international travellers from five travel clinics across the United States between 2017 and 2020.^8^^,^^9^ Travellers self-collected stool samples immediately before and after travel.^9^ We utilized an internally validated culture-based screening protocol followed by phenotypic ESBL confirmatory testing, as previously described, to detect presence of ESBL-PE.^22^ We administered pre- and post-travel questionnaires to identify medical/health characteristics, activities and behaviour before and during international travel, along with destination and trip duration data. This current report was not the main rationale for study initiation, so we did not perform any a priori study size calculations. We excluded travellers if they were missing data for detection of ESBL before or after travel, or if they were ESBL-PE-positive before travelling (Figure 1A). We defined acquisition of ESBL-PE as a traveller with a negative culture for ESBL-PE prior to travel, and a positive culture upon first follow-up after return. If individuals were positive for ESBL-PE upon return, we followed up with them at 90 and 180 days to test again for ESBL-PE-producing organisms. Institutional review board approval was obtained from the human research committee at each participating enrolment site.

### Data cleaning and candidate feature selection

We followed guidelines from the Transparent Reporting of a multivariable prediction model for Individual Prognosis Or Diagnosis checklist (Supplemental Checklist).^23^ All data processing and analyses were performed in R version 4.1.0. Detailed descriptions of data processing (and utilized R packages) are found in the supplemental methods.

We selected candidate features based on clinical expertise and past identification in published literature, pulling features from traveller questionnaires and multiple external databases. We included the gross domestic product based on purchasing power parity (GDP-PPP) of each traveller’s unique destination(s) by averaging WorldBank data from 1990 to 2020 (Supplemental Table 1). We also incorporated destination country(ies) rankings for (i) waste management and (ii) sanitation and drinking water from the 2020 Yale Environmental Performance Index (EPI) (Supplemental Table 2).^24^ These two metrics were chosen because they relate to potential exposure to enteric organisms. We excluded 22 individuals who travelled to destinations not included in the GDP-PPP listing and/or Yale EPI ranking. We calculated mean daily temperature during travel from the National Centers for Environmental Information’s Global Summary of the Day (GSOD), using average daily temperature data from stations <50 km from each country’s capital (or other populous areas if stations were not within 50 km of the capital) (https://www.ncei.noaa.gov/access/metadata/landing-page/bin/iso?id=gov.noaa.ncdc:C00516). We used capital city information as these are often populous areas that may attract travellers. We used the *gsodr* package to import all GSOD temperature data.^25^ We also included travel region risk of acquisition, using results from the carriage of multiresistant bacteria after travel (COMBAT) study, a multicentre cohort study that determined the proportion of Dutch travellers who acquire ESBL-PE during travel (Supplemental Table 3).^4^ The mean proportion of ESBL-PE acquisition for each WHO region was used as the probability of acquisition for each traveller. If an individual travelled to multiple regions/countries, each of these destination features was weighted by time in each region/country.

The list of 27 included features and the complete list of all features available can be found in Supplemental Table 4 and Supplemental Table 5, respectively. All features were centred and scaled utilizing the *caret* package.^26^ One traveller was missing two (7.7%) features, whereas five were missing one (3.8%). With such limited missing data, we performed a single round of imputation to replace missing features, utilizing the *mice* package.^27^

### Identifying predictive features, model fitting and performance

To model features predictive of ESBL acquisition, we utilized the *glmnet* R package to perform LASSO regression (α = 1) as a screening step to select predictive features.^28^ We used the *glmnet* function to select features, utilizing the minimum lambda value at the specific degrees of freedom that corresponded to desired number of features (1–15). To demonstrate direction of effect, we fit a multivariable logistic regression (LR) to the dataset, as performed previously.^29^ We used the *glm* function, adding all LASSO-selected terms, to calculate coefficients, error, adjusted odds and *P* values.

We performed repeated cross-validation using 80% training and 20% testing splits (100 iterations) to estimate the generalizable performance of the model fits (Figure 1B). As described earlier, we first selected features with LASSO regression on the training set. We then ran either a random forest (RF) (using the *ranger* package) with 500 trees (and other default settings), or a LR model (using *glm*) on the selected features.^30^ We then tested the models on the 20% testing split. We calculated the cvAUC in each iteration using the cvAUC package (95% confidence intervals were calculated with *ci.cvAUC*) and calculated calibration intercept and slope using logistic models as performed previously.^31^ We used the *roc* and *coords* functions from the *pROC* package to calculate sensitivity, specificity, positive predictive value and negative predictive value.

For model performance at 90 and 180 days post return, we filtered subjects with data at said timepoints (*n* = 523 at 90 days; *n* = 512 at 180 days) and ran our model training on ESBL positivity at immediate return, but tested on ESBL positivity at either return or the later timepoint (Day 90 or 180). By re-running the models at the selected time-points, we could better compare model performance at the different follow-up dates.

## Results

### Dataset description

A cohort of 528 travellers satisfied our inclusion criteria. Of these individuals, 200 (37.8%) returned from travel as ESBL-PE-positive. Demographic and other characteristics of these travellers (total and ESBL-PE-positive) are outlined in Table 1 and in previous publications.^8^^,^^9^ Travellers ranged in age from 1 to 81 years old, were predominantly female, and travelled between 3 and 161 days. Participants travelled to all WHO regions, with the majority travelling to Africa, the Americas (outside of the US) or Southeast Asia. In total, 104 countries were documented as destinations, spanning all continents except for Antarctica. Half (50.4%) of the participants reported diarrhoea (mild, moderate or severe) during travel.

**Table 1:** Characteristics of analysed cohort

Column1	Overall (*n* = 528)	Acquired ESBL (*n* = 200)
Sex		
Female	317 (60.0%)	109 (34.4%)^a^
Male	211 (40.0%)	91 (43.1%)^a^
Age (years)		
Mean (SD)	48.5 (17.8)	48.4 (17.7)
Median (min, max)	51 (1.4, 81)	51.5 (1.4, 81)
Trip duration (days)		
Mean (SD)	19.1 (20.0)	21.0 (24.0)^a^
Median (min, max)	14 (3, 161)	15 (4, 161)^a^
Travel region (WHO)		
Africa	237 (44.9%)	90 (38.0%)^a^
America	152 (28.8%)	45 (30.0%)^a^
Southeast Asia	113 (21.4%)	56 (50.0%)^a^
Western Pacific	64 (12.1%)	22 (34.4%)^a^
Eastern Mediterranean	28 (5.3%)	10 (35.7)^a^
Europe	22 (4.2%)	8 (36.3%)^a^
Any diarrhoea		
Yes	266 (50.4%)	122 (45.9%)^a^
No	262 (49.2%)	78 (29.8%)^a^

^a^Percentage values calculated row-wise.

### Model cross-validation and performance

**Figure 2:**
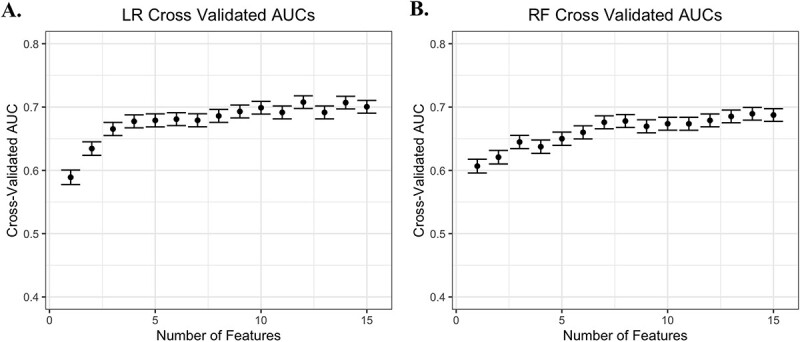
Receiver operating characteristic cvAUCs for either LR (A) or RF (B) models with 1–15 features. Graphs show mean cvAUC for 100 iterations, error bars represent 95% CIs

We performed cross-validation using LASSO regression for feature selection, followed by modelling with RF or LR models (Figure 1B). Increasing the number of features increased the cross-validated area under the receiver operating characteristic curve (cvAUC) of each model (Figure 2). In the LR model, we observed the slope begin to decrease when around four features were included, with a cvAUC of 0.68 (95% CI 0.67–0.69) (Table 2). Increasing the LR model to around 10 features resulted in a near-maximum cvAUC of 0.70 (95% CI 0.69–0.71). Modelling with LR outperformed modelling with RF, in which we observed cvAUCs of 0.67 (95% CI 0.66–0.68) in a 10-feature model and 0.63 (95% CI 0.62–0.64) in a 4-feature model.

**Table 2:** Model performance

Statistic	10-Feature LR (95% CI)	10-Feature RF (95% CI)	4-Feature LR (95% CI)	4-Feature RF (95% CI)
cvAUC mean	0.70 (0.69–0.71)	0.67 (0.66–0.68)	0.68 (0.67–0.69)	0.63 (0.62–0.64)
Calibration intercept	−0.04 (−0.09 to 0.02)	−0.05 (−0.09 to 0.00)	0.04 (−0.01 to 0.09)	−0.03 (−0.08 to 0.02)
Calibration slope	0.78 (0.71–0.84)	0.63 (0.59–0.67)	0.82 (0.76–0.88)	0.51 (0.47–0.54)
Sensitivity 0.70				
Specificity	0.58 (0.56–0.59)	0.56 (0.55–0.57)	0.55 (0.54–0.56)	0.52 (0.51–0.53)
Positive predictive value	0.51 (0.50–0.52)	0.50 (0.49–0.51)	0.49 (0.48–0.50)	0.47 (0.46–0.48)
Negative predictive value	0.75 (0.74–0.76)	0.75 (0.74–0.75)	0.75 (0.74–0.75)	0.74 (0.73–0.75)
Sensitivity 0.80				
Specificity	0.46 (0.44–0.47)	0.46 (0.44–0.47)	0.44 (0.43–0.45)	0.41 (0.40–0.43)
Positive predictive value	0.48 (0.47–0.49)	0.48 (0.47–0.49)	0.47 (0.46–0.48)	0.45 (0.44–0.46)
Negative predictive value	0.78 (0.77–0.79)	0.78 (0.77–0.79)	0.78 (0.77–0.78)	0.77 (0.76–0.78)
Sensitivity 0.90				
Specificity	0.31 (0.29–0.32)	0.32 (0.31–0.33)	0.30 (0.29–0.31)	0.28 (0.27–0.29)
Positive predictive value	0.45 (0.43–0.45)	0.45 (0.44–0.46)	0.44 (0.43–0.45)	0.43 (0.42–0.44)
Negative predictive value	0.82 (0.81–0.83)	0.83 (0.82–0.84)	0.82 (0.81–0.83)	0.82 (0.81–0.83)

A clinical decision-support tool for detection of ESBL acquisition in returning travellers would likely benefit from increased sensitivity, with less regard for specificity. Our rationale is that treatment with an antibiotic that unnecessarily targets ESBL-PE (e.g. a fourth-generation cephalosporin, a carbapenem or inclusion of a beta lactamase inhibitor) would do less harm than erroneous treatment with an ineffective antibiotic during a moderate to severe infection. Therefore, we analysed LR and RF performance at sensitivities of 0.70, 0.80 and 0.90 (Table 2). At a sensitivity of 0.70, the 10-feature LR model performed with a specificity of 0.58 (95% CI 0.56–0.59), suggesting that correctly classifying 70% of positive cases can be done while positively classifying almost 60% of negative cases. Though the 10-feature LR has higher specificity than the four-feature LR at a sensitivity of 0.70, the specificity is similar between the two at a sensitivity of 0.90. At high sensitivity, all models contained similar positive predictive values (PPVs) (0.43–0.44 at sensitivity of 0.90) and similar negative predictive values (NPVs) (0.82–0.83 at sensitivity of 0.90).

We estimated model calibration slope and intercepts as well (Table 2). In all models, calibration intercept was near zero. In all but the four-feature LR, intercepts were slightly negative, suggesting a slight tendency to overestimate risk of acquisition. The four-feature LR had the best calibration slope (closest to one). However, all slopes were slightly below one, suggesting the probabilities predicted are underconfident.

Because travellers may not present with infection immediately after return, we ran our model again, still training on ESBL positivity upon return, but now testing on ESBL positivity 90 or 180 days after return. The LR models tested on positivity at these dates performed similarly to models tested at the time of return (Supplemental Figure 1). Importantly, individuals who remain colonized at Days 90 or 180 have significantly higher risk scores than those who are no longer colonized at these timepoints (Supplemental Figure 2). This further suggests that this tool also is useful in predicting maintained colonization.

### Model selection of predictive features and association with acquisition

We utilized LASSO regression on the entire dataset to select the features most predictive of ESBL acquisition (Supplemental Table 6). When modelling with four features, the model selected: antibiotics for traveller’s diarrhoea, Yale EPI waste management rankings, COMBAT-derived regional probabilities and any diarrhoea as predictive features. When increasing the number of features to 10, the model selected the previously mentioned features, and two other gastrointestinal-(GI)-associated features: having normal bowel movement upon return, and probiotic usage prior to or during travel. The model also selected trip characteristics including reasons for travel and rural destination as predictive features. The model also selected traveller sex. Interestingly, Yale EPI water and sanitation rankings, and GDP-PPP of destination were not selected as informative for risk of acquisition in this model.

**Figure 3:**
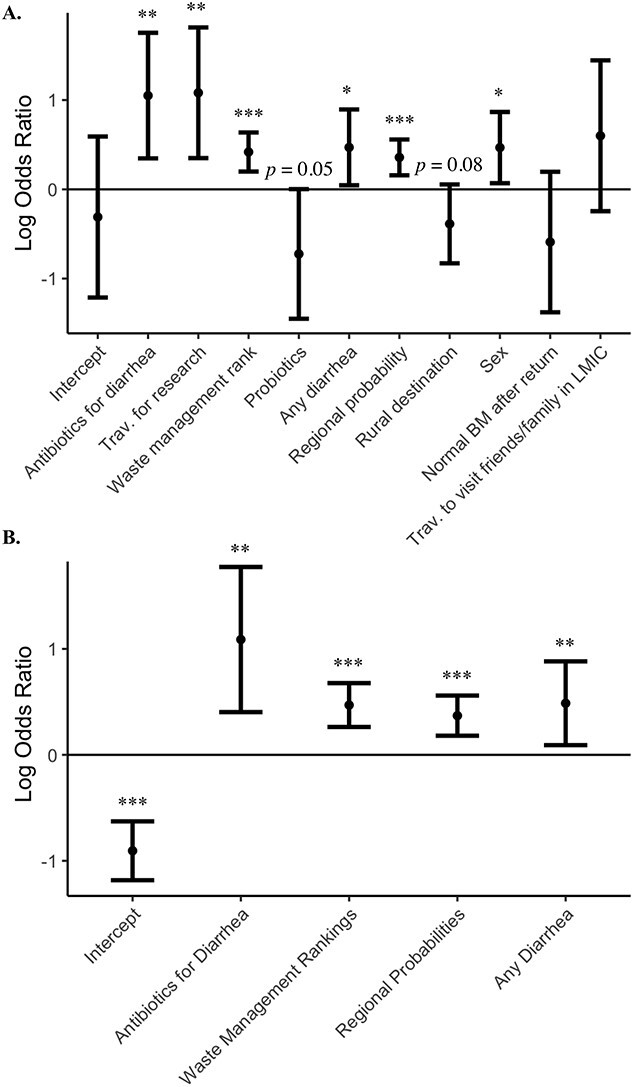
Log odds ratios (with 95% CI) of models with either 10 features (A) or four features (B) to demonstrate directionality of effect. Modelled on the whole dataset (*n* = 528) and the LASSO-selected features. ^*^P < 0.5, ^*^^*^P < 0.01, ^*^^*^P < 0.001

Although our goal was for prediction and not causal inference, the directionality of these predictive features from multivariable LR is detailed in Figure 3. In the four-feature model, we found that having diarrhoea (and taking antibiotics for diarrhoea) while travelling (*P* = 0.016, *P* = 0.002, respectively) were significantly associated with increased risk. We also found that travelling to regions with higher regional acquisition rate probabilities (*P* = 0.001) and travelling to countries with worse Yale EPI rankings for waste management (*P* < 0.001) were associated with higher risk. We observed the same trend in the 10-feature model (*P* = 0.030, *P* = 0.003, *P* < 0.001, *P* < 0.001, respectively), but also found that travelling for research purposes (*P* = 0.003) and male sex (*P* = 0.022) were associated with increased risk. We conversely observed that probiotics usage before and/or during travel was trending towards significance (*P* = 0.05), along with travel to a rural destination (*P* = 0.09).

## Discussion

International travellers are at risk of ESBL-PE acquisition during time abroad. Infections with ESBL-PE are associated with increased disease severity, potentially due to less effective treatment.^14^^,^^32^ Distinct from GI colonization, international travel may increase risk for extraintestinal infection with ESBL-PE.^33^ Relatedly, GI carriage of ESBL-PE is associated with an increased occurrence of subsequent ESBL-PE bloodstream infection.^34^ Numerous studies have identified risk factors for ESBL-PE acquisition among international travellers, but prediction models to inform a CPR for identification of ESBL-PE are lacking. Tools that help predict risk of returning with ESBL-producing organisms can help guide treatment options in the event of infection after travel. Here, we describe our derivation and internal validation of a prediction model of ESBL-PE acquisition in returning international travellers that incorporates both traveller- and destination-specific characteristics. Our model with four features can be implemented by asking travellers three general questions: destination(s), diarrhoea during travel and use of antibiotics for any diarrhoea.

Our 10-feature model selected multiple gastrointestinal-related features previously associated with ESBL-PE acquisition.^3^^,^^6^^,^^7^^,^^9^^,^^17^^,^^35^ However, by including destination features in our modelling, we found several additional predictive features, including destination region, rural vs urban travel and waste management ranking of destination country. Travel destination region is linked to risk of ESBL-PE acquisition, with travel to Southeast Asia specifically associated with higher risk.^17^ Although not previously studied in international travellers, endemic ARO carriage is inversely correlated with GDP per capita and infrastructure.^36^ Temperature is associated with resistance in multiple pathogens, but we only observed this feature selected as predictive when modelling with 15 features (data not shown).^37^ Our inclusion of novel location-specific parameters demonstrates their potential importance for travel-related prediction models.

A CPR identifying risk of ESBL-PE acquisition has potential applications both before and after international travel. Based on the features we identified with high predictive importance, travellers can be counselled on the potential magnitude of risks for ARO acquisition. Pre-travel education efforts aimed at addressing risk factors for acquisition may include reduction of risk factors for traveller’s diarrhoea, and appropriate antibiotic use for treatment of traveller’s diarrhoea. Our model may be useful for clinicians if implemented as a decision support tool. Bacterial culture with organism identification and antimicrobial susceptibility often requires multiple days for finalization. While awaiting definitive results for a clinically infected patient, clinicians often initiate broad coverage with agents such as beta-lactams. For antimicrobial stewardship purposes, clinicians do not typically target ESBL organisms unless the patient has known risk factors or clinical suspicion of ESBL acquisition, or ESBL-producing organisms are isolated on culture. Identification of those at high risk for ESBL colonization could direct clinicians to select initial ESBL-targeting antibiotic regimens such as carbapenems which demonstrate improved coverage of ESBL organisms in the setting of undifferentiated infection.

Alternatively, identification of asymptomatic colonization may be of interest to inform infection control precautions in high-risk settings.^32^^,^^38^ GI carriage of ESBL-PE is a risk factor for transmission or possible infection.^4^^,^^34^ ESBL-producing *Klebsiella* has been associated with the risk of intensive care unit acquisition through patient-to-patient contact,^39^ and large-scale outbreaks from ESBL *E. coli* have also been observed.^32^^,^^40^ A CPR predicting ESBL-PE may thus help to improve both patient care and infection control practices (e.g. preemptive isolation upon admission). Based on our findings, it may be reasonable to screen or preemptively isolate returning travellers with high risk for ESBL-PE acquisition, especially in the context of immunocompromised or other high-risk populations. The decision between screening with stool culture versus preemptive isolation would be determined by individual hospital epidemiology protocols.

We report results of models with a fixed high sensitivity, at the expense of specificity. With decreased specificity, PPV values are decreased, indicating that this tool is more useful when a traveller is predicted to be negative, than if predicted positive. PPV/NPV values depend on the prevalence of the outcome in a dataset, and the prevalence of ESBL acquisition in our cohort is within the range of prior studies.^3–9^ However, when subsetting cohorts by region of travel, model recalibration might be necessary. With appropriate external validation and potential recalibration, this prediction model could be built into an electronic tool (potentially through a web or mobile application) that can be utilized by medical practitioners, as our group has done previously.^41^

### Limitations

Our best model, a 10-feature LR, might be too burdensome and may discourage downstream use by clinicians. However, the four-feature LR performs close to the maximum achievable AUC. Future work with larger sample sizes could increase the predictive ability of our modelling, while maintaining a small number of included features. Another limitation is that our destination information was limited to the country level, potentially decreasing the usefulness of destination-related features in geographically or socioeconomically heterogeneous countries. Future prediction applications could benefit from more accurate population density, sanitation-related and weather-related features experienced during travel by utilizing more granular travel data (potentially through cell phone location data). Future work will include the validation and re-derivation of this tool after integrating more informative external databases dependent on more precise tracking data. As some subregions (such as Northern Africa and Western Asia) did not have large numbers of travellers, applicability of this model may be limited in travellers to those areas. After the collection of larger training sets and external validation, we will have a better understanding of model performance in travellers to those destinations.

We also excluded individuals who were ESBL-PE-positive before travel. In limiting our prediction to that of new acquisition, we do not consider individuals who acquire new ESBL strains during travel, nor those who gain and lose ESBL-PE before returning home.^42^ Relatedly, ESBL-PE present at low-levels could have been missed during pre-travel testing. It is possible that those organisms bloom during travel, obfuscating our definition of acquisition.

We have yet to externally validate the model derived in our study, thus limiting our understanding of its potential generalizability and reproducibility. Due to differing prevalences in other cohorts, or changing rates of ESBL-PE over time, we may need to recalibrate our models before practical application. However, if the model achieves similar performance metrics on external validation, it will perform similarly to the Centor criteria. The Centor criteria is a widely used tool (commonly cited in clinical guidelines and used in diagnostic and treatment algorithms of large health systems) that guides testing decisions for group A streptococcal pharyngitis in outpatient settings.^43^^,^^44^ Relatedly, this work was performed on travellers from the US, and the data should be confirmed in travellers from other nations. Data used in our model development were collected before the start of the COVID-19 pandemic; changes in travel patterns, behaviour and hygiene measures related to COVID-19 may affect the utility of our model. Finally, this work did not address susceptibility to other antibiotics, such as fluoroquinolones. Therefore, the benefit of switching antibiotic class in response to this model has not been fully evaluated.

## Conclusion

Overall, our work illustrates the possibility of combining traveller-specific and destination-specific data to estimate the risk of ESBL-PE acquisition in international travellers. Further validation and implementation of this model may help to identify returning travellers with potential colonization with ESBL-PE who may benefit from more appropriate empiric antimicrobial selection.

## Funding

This work was supported by the Centers for Disease Control and Prevention [U01CK000490, U01CK000633]; the National Institute of Allergy and Infectious Diseases, National Institutes of Health, Department of Health and Human Services [U19AI110818, R01AI135114, R01HD102540]; the National Institutes of Health Ruth L. Kirschstein National Research Service Award [T32HG008962] from the National Human Genome Research Institute; and TRIAD, with funding in part from the National Center for Advancing Translational Sciences of the National Institutes of Health [UL1TR002538]. The content is solely the responsibility of the authors and does not necessarily represent the official views of the National Institutes of Health.

## Author Contributions

D. Brown (Formal analysis-Lead, Funding acquisition-Supporting, Methodology-Lead, Visualization-Lead, Writing—original draft-Lead, Writing—review & editing-Equal), Colin Worby (Data curation-Equal, Resources-Equal, Writing—review & editing-Equal), Melissa Pender (Writing—original draft-Supporting, Writing—review & editing-Equal), Ben Brintz (Formal analysis-Supporting, Methodology-Supporting, Resources-Equal, Writing—review & editing-Equal), Edward Ryan (Data curation-Equal, Writing—review & editing-Equal), Sushmita Sridhar (Data curation-Equal, Writing—review & editing-Equal), Elizabeth Oliver (Data curation-Equal), Jason Harris (Data curation-Equal, Writing—review & editing-Equal), Sarah Turbett (Data curation-Equal, Writing—review & editing-Equal), Sowmya Rao (Data curation-Equal, Writing—review & editing-Equal), Ashlee Earl (Data curation-Equal, Writing—review & editing-Equal), Regina LaRocque (Conceptualization-Equal, Data curation-Equal, Funding acquisition-Equal, Project administration-Equal, Writing—review & editing-Equal), Daniel Leung (Conceptualization-Equal, Funding acquisition-Equal, Methodology-Supporting, Project administration-Equal, Resources-Equal, Supervision-Equal, Writing—original draft-Supporting, Writing—review & editing-Equal).

## Data availability statement

All code and data is available at https://github.com/dgbrow02/esbl_prediction_code and in the supplementary methods.

## Conflicts of interest

S.E.T. has received royalties from UpToDate. R.C.L. receives payments for editorial services from CDC Foundation and from UpToDate.

## Supplementary Material

Supplemental_tables_taad028

20230224_combined_supplement_taad028
