# Supplementary material for: Development of a prediction model for the acquisition of extended spectrum beta-lactam-resistant organisms in U.S. international travellers
Source: J Travel Med. 2023 Mar 2;30(6):taad028. doi: 10.1093/jtm/taad028 (PMC10628771; doi:10.1093/jtm/taad028)
Supplement: 20230224_combined_supplement_taad028 [file 20230224_combined_supplement_taad028.pdf]

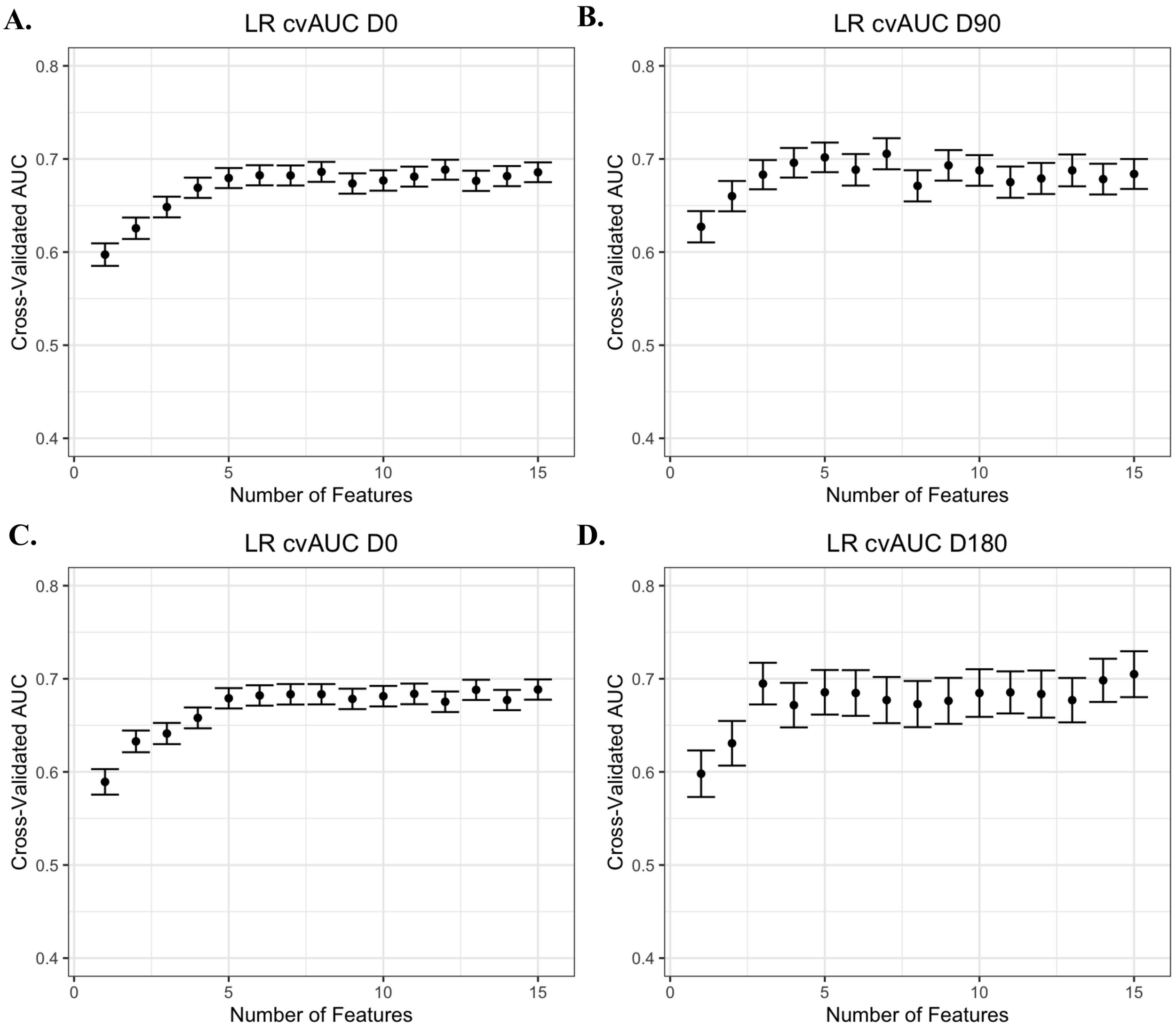

Supplemental Figure 1. cvAUC values from LR models trained on the top 1–15 features to predict ESBL positivity at return timepoint but tested at different timepoints. A, B depict the cohort that was tested at both day 0 and, if positive, day 90 (n = 523). If negative at day 0, subjects were assumed negative at day 90. A. cvAUC predicting positivity at day 0. B. cvAUC predicting positivity at day 90. C, D depict the cohort from A,B that were tested again at day 180, if positive (n = 512). Subjects were assumed negative if tested negative at either day 0 or 90. Testing ESBL positivity at day 0 (C), or day 180 (D).

A.

B.

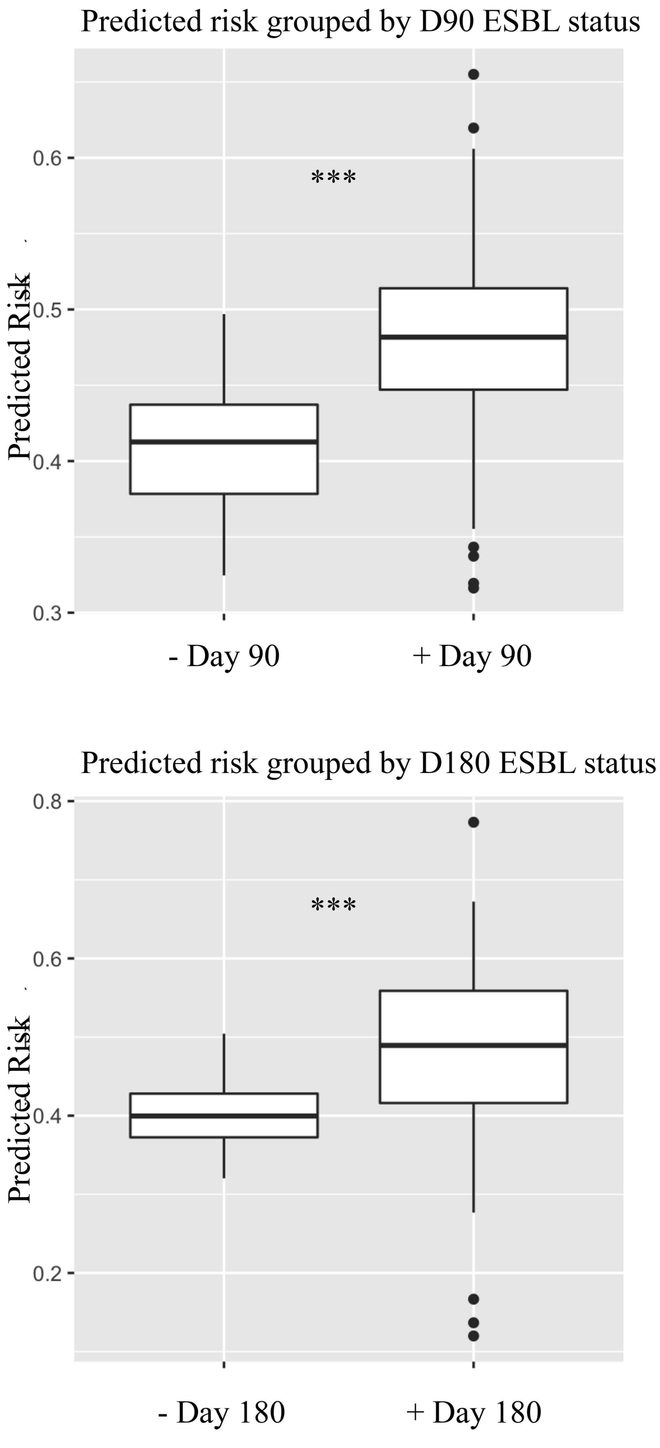

Supplemental Figure 2. Boxplots illustrating means and quartiles of risk scores of initially positive subjects grouped by status at future timepoints. A. depicts the risk scores of all ESBL+ individuals in testing cohorts, grouped by ESBL status at day 90. B. depicts the risk scores of all ESBL+ individuals in testing cohorts, grouped by ESBL status at day 180. ***p<0.001 by logistic regression.

Supplementary Data

Additional Methods

Feature selection process

To not provide the model with too many candidate features (to avoid overfitting), we selected features previously identified as associated with ESBL acquisition [1-9]. We specifically selected features relating to diarrhea, antibiotic consumption, reason for travel, and destination-specific characteristics. We excluded all features from inclusion that were missing over 40% of the responses. We also required any selected feature to consist of more than 5% positive, non-zero answers. Though we collected many metrics of diarrhea, to limit the number of features, we merged the features, diarrhea (which corresponds to three or more loose stools in a single day) and mild diarrhea, into a single feature, any diarrhea. We deemed a positive response to either feature as a positive for "any diarrhea." Similarly, we collected detailed information on antibiotics utilize for traveler's diarrhea. We did not include each antibiotic, only general use of antibiotics for diarrhea. If a value was reported as positive for any class of antibiotic taken for diarrhea (ciprofloxacin, azithromycin, rifaximin, or other), we dictated the subject as positive for "antibiotics for diarrhea."

We included the feature, "resolution of abnormal bowel movements after return". For individuals who did not report diarrhea during travel, we assigned a value of 1 (i.e. equivalent to bowel movement returning to normal in those that reported diarrhea). Similarly, we tracked if travel companions reported diarrhea or not. We assigned a value of 0 for those who did not report travel companions (i.e. equivalent to having a travel companion that did not have diarrhea).

During processing, one individual return date was chronologically before their departure date. As the written return date was 01/10/2019, and the written departure was 12/11/2019, we assumed the return was 2020, and used this value instead.

Location-specific data sources

We calculated all destination features utilizing reported time in each country. On a few occasions, a subject reported traveling to a single country, and reported total trip length, but not time in that specific country. In these situations, we assumed the time spent in that country was equal to total trip length.

Some individuals reported destination country names as non ISO-2 codes. Two individuals traveled to three reported countries: "FRITCH," "ARCL," and "THKHLABT." For these individuals, we assigned the first country visited as FR (France), the second as AR (Argentina), and the third as TH (Thailand). One traveler reported the third country visited as "ITESPTMC," which we assigned as IT (Italy). One traveler reported a country as "OTA," which we assigned to "IT."

We included external information relating to destination country characteristics and weather experienced during travel as additional candidate features. We extracted gross domestic product based on purchasing power parity (GDP-PPP) from the World Bank website, (www.data.worldbank.org/indicator/NY.GDP.MKTP.PP.CD, **Supplemental Table 1**) which contains annual GDP-PPP of up to 246 countries, regions, and territories from 1990 to 2020. We calculated the mean values for each country and matched these data to the destination of each traveler. If a traveler spent time in multiple countries, we calculated the mean GDP of all visited countries, weighted by time spent in each country. We incorporated two metrics from the 2020 Yale Environmental Performance Index, a comprehensive analysis ranking countries by

environmental health and sustainability [10]. Specifically, we incorporated the country rankings for 1) waste management and 2) sanitation and drinking water and applied them to each traveler (**Supplemental Table 2**). These two metrics were chosen because they directly relate to exposure to enteric organisms. If a traveler visited multiple countries, we calculated a time-weighted mean for that traveler. We excluded 22 individuals who traveled to destinations not included in the GDP-PPP listing and/or Yale EPI ranking. These destinations included Aruba, Bermuda, British Virgin Islands, Caribbean Netherlands (Bonaire, Sint Eustatius, and Saba), Cuba, Curacao, Hong Kong, Macao, Venezuela, and Virgin Islands. If a traveler visited multiple countries, any excluded destinations were omitted from analysis, but the data for the included countries kept.

Mean temperature during travel was estimated using the National Centers for Environmental Information's Global Summary of the Day and the *gsodr* r package [11]. For each country by date visited in our dataset, we averaged the weather data from stations less than 50 km from the country capital. We chose capital cities as these are often areas where individuals may visit while traveling. For Belize and Malawi, we used a weather center reference point of Belize City and Blantyre, respectively, because no GSOD weather stations are within 50 km of these countries' capitals. These cities, however, exist as populous areas near GSOD weather stations. We used the *nearest_stations* function to find all stations within 50 KM of each capital coordinates. When then used *get_GSOD* to import weather data for the selected stations. We summarized the mean daily temperatures for the stations selected in each country. We then averaged this country-specific daily temperature for each day of each persons' travel.

Data processing

To improve model performance, we centered and scaled all 27 features with the *preProcess* command from the caret (classification and regression training) package. The caret package was designed to aid in the creation of prediction models [12]. To impute missing data, we utilized the mice (multivariate imputation by chained equations) package [13]. We specified the prediction of missing features by utilizing the logistic regression (logreg) method. We chose this method as all missing features were categorical. As missing data was rare, we generated values with a single round of imputation.

Modeling

Both LR and RF models utilize the same variable selection procedure. The model splits the data into 80% training and 20% testing sets. On the training set, we ran *glmnet* (from the eponymous package), on the entire training set, utilizing a series of lambda values that would select 1-15 degrees of freedom in the generated models [14]. We set α=1, to perform LASSO regression, specifically. We then use the *VarImp* function, selecting the minimum lambda value corresponding to the number of features we desired in our model. Using these selected features, we trained the model with *glm* (to run logistic regressions) or *ranger* (to run random forests) on the training set. We used the *predict* function to test these models on the hold-out testing sets. This process was repeated 100 times for each model at each number of features. We specifically utilized logistic regression or random forest as these are tools with broad applicability to prediction work [15, 16]. Logistic regression allows the estimation of maximum likelihood using combination of features. Random forest is an ensemble learning approach that averages iterations of decision trees that each determines the most accurate method to discriminate between groups.

Calculating calibration intercept and slope

We calculated intercept by modeling the logistic regression Y = α + LP, where Y is the known outcome, LP is the logit estimate (utilized as an offset term) and α the coefficient. Slope was calculated by determining the slope after modeling the logistic function $Y = \beta_0 + \beta_1 * LP$, where β_1 is the slope [17].

1. Kantele A, Laaveri T, Mero S, et al. Antimicrobials increase travelers' risk of colonization by extended-spectrum betalactamase-producing Enterobacteriaceae. Clin Infect Dis **2015**; 60(6): 837-46.
2. Arcilla MS, van Hattem JM, Haverkate MR, et al. Import and spread of extended-spectrum β-lactamase-producing Enterobacteriaceae by international travellers (COMBAT study): a prospective, multicentre cohort study. The Lancet Infectious Diseases **2017**; 17(1): 78-85.
3. Woerther PL, Andremont A, Kantele A. Travel-acquired ESBL-producing Enterobacteriaceae: impact of colonization at individual and community level. J Travel Med **2017**; 24(suppl_1): S29-S34.
4. Flateau C, Duron-Martinaud S, Haus-Cheymol R, et al. Prevalence and risk factors for Extended-Spectrum Beta-Lactamase-producing- Enterobacteriaceae in French military and civilian travelers: A cross-sectional analysis. Travel Med Infect Dis **2018**; 23: 44-8.
5. Sridhar S, Turbett SE, Harris JB, LaRocque RC. Antimicrobial-resistant bacteria in international travelers. Curr Opin Infect Dis **2021**; 34(5): 423-31.
6. Mellon G, Turbett SE, Worby C, et al. Acquisition of Antibiotic-Resistant Bacteria by U.S. International Travelers. N Engl J Med **2020**; 382(14): 1372-4.
7. Worby CJ, Earl AM, Turbett SE, et al. Acquisition and Long-term Carriage of Multidrug-Resistant Organisms in US International Travelers. Open Forum Infect Dis **2020**; 7(12): ofaa543.
8. Collignon P, Beggs JJ, Walsh TR, Gandra S, Laxminarayan R. Anthropological and socioeconomic factors contributing to global antimicrobial resistance: a univariate and multivariable analysis. The Lancet Planetary Health **2018**; 2(9): e398-e405.
9. MacFadden DR, McGough SF, Fisman D, Santillana M, Brownstein JS. Antibiotic Resistance Increases with Local Temperature. Nat Clim Chang **2018**; 8(6): 510-4.
10. Wendling ZA, Emerson, J. W., de Sherbinin, A.,, Esty DC, et al. . 2020 Environmental Performance Index. New Haven, CT: Yale Center for Environmental
Law & Policy: Yale, **2020** 2029.
11. Sparks AH, T; Nelson, A GSODR: Global Summary Daily Weather Data in R. The Journal of Open Source Software **2017**; 2(10).
12. Kuhn M. Building Predictive Models in R Using the caret Package. Journal of Statistical Software **2008**; 28(5): 1-26.
13. van Buuren SG-O, K. mice: Multivariate Imputation by Chained Equations in R. Journal of Statistical Software **2011**; 45(3): 1-67.
14. Friedman JH, T; Tibshirani, R Regularization Paths for Generalized Linear Models via Coordinate Descent. Journal of Statistical Software **2010**; 33(1): 1–22.

15. Brintz BJ, Haaland B, Howard J, et al. A modular approach to integrating multiple data sources into real-time clinical prediction for pediatric diarrhea. Elife **2021**; 10.
16. Brintz BJ, Howard JI, Haaland B, et al. Clinical predictors for etiology of acute diarrhea in children in resource-limited settings. PLoS Negl Trop Dis **2020**; 14(10): e0008677.
17. Van Calster B, McLernon DJ, van Smeden M, et al. Calibration: the Achilles heel of predictive analytics. BMC Med **2019**; 17(1): 230.

S1 Checklist: TRIPOD Checklist: Prediction Model Development

Section/Topic	Item	Checklist Item	Page
**Title and abstract**			
Title	1	Identify the study as developing and/or validating a multivariable prediction model, the target population, and the outcome to be predicted.	1
Abstract	2	Provide a summary of objectives, study design, setting, participants, sample size, predictors, outcome, statistical analysis, results, and conclusions.	3
**Introduction**			
Background and objectives	3a	Explain the medical context (including whether diagnostic or prognostic) and rationale for developing or validating the multivariable prediction model, including references to existing models.	5-6
	3b	Specify the objectives, including whether the study describes the development or validation of the model or both.	6
**Methods**			
Source of data	4a	Describe the study design or source of data (e.g., randomized trial, cohort, or registry data), separately for the development and validation data sets, if applicable.	6-7
	4b	Specify the key study dates, including start of accrual; end of accrual; and, if applicable, end of follow-up.	6
Participants	5a	Specify key elements of the study setting (e.g., primary care, secondary care, general population) including number and location of centres.	6
	5b	Describe eligibility criteria for participants.	6-7
	5c	Give details of treatments received, if relevant.	N/A
Outcome	6a	Clearly define the outcome that is predicted by the prediction model, including how and when assessed.	6
	6b	Report any actions to blind assessment of the outcome to be predicted.	NA
Predictors	7a	Clearly define all predictors used in developing or validating the multivariable prediction model, including how and when they were measured.	7-8
	7b	Report any actions to blind assessment of predictors for the outcome and other predictors.	6
Sample size	8	Explain how the study size was arrived at.	7
Missing data	9	Describe how missing data were handled (e.g., complete-case analysis, single imputation, multiple imputation) with details of any imputation method.	8
Statistical analysis methods	10a	Describe how predictors were handled in the analyses.	7-9
	10b	Specify type of model, all model-building procedures (including any predictor selection), and method for internal validation.	8-9
	10d	Specify all measures used to assess model performance and, if relevant, to compare multiple models.	9
Risk groups	11	Provide details on how risk groups were created, if done.	N/A
**Results**			
Participants	13a	Describe the flow of participants through the study, including the number of participants with and without the outcome and, if applicable, a summary of the follow-up time. A diagram may be helpful.	10
	13b	Describe the characteristics of the participants (basic demographics, clinical features, available predictors), including the number of participants with missing data for predictors and outcome.	8,10
Model development	14a	Specify the number of participants and outcome events in each analysis.	10
	14b	If done, report the unadjusted association between each candidate predictor and outcome.	N/A
Model specification	15a	Present the full prediction model to allow predictions for individuals (i.e., all regression coefficients, and model intercept or baseline survival at a given time point).	12; ST6

	15b	Explain how to the use the prediction model.	12
Model performance	16	Report performance measures (with CIs) for the prediction model.	10-11
**Discussion**			
Limitations	18	Discuss any limitations of the study (such as nonrepresentative sample, few events per predictor, missing data).	15-16
Interpretation	19b	Give an overall interpretation of the results, considering objectives, limitations, and results from similar studies, and other relevant evidence.	13-14
Implications	20	Discuss the potential clinical use of the model and implications for future research.	14-15
**Other information**			
Supplementary information	21	Provide information about the availability of supplementary resources, such as study protocol, Web calculator, and data sets.	S1
Funding	22	Give the source of funding and the role of the funders for the present study.	18

We recommend using the TRIPOD Checklist in conjunction with the TRIPOD Explanation and Elaboration document.
